# Health-Related Quality of Life after Ischemic Stroke: The Impact of Pharmaceutical Interventions on Drug Therapy (Pharmaceutical Care Concept)

**DOI:** 10.1186/1477-7525-8-59

**Published:** 2010-06-18

**Authors:** Carina Hohmann, Roland Radziwill, Juergen M Klotz, Andreas H Jacobs

**Affiliations:** 1 Klinikum Fulda gAG, Department of Neurology, Pacelliallee 4, 36043 Fulda, Germany; 2 Klinikum Fulda gAG, Department of Pharmacy and Patient Counselling, Pacelliallee 4, 36043 Fulda, Germany; 3 European Institute for Molecular Imaging - EIMI, University of Muenster, Technologiehof L1, Mendelstrasse 11, 48149 Muenster, Germany

## Abstract

**Background:**

Health-related quality of life (HRQoL) after stroke is an important healthcare measure. Pharmaceutical Care (PC) is an evolving concept to optimize drug-therapy, minimize drug-related problems, and improve HRQoL of patients. The purpose of this study was to evaluate the impact of PC on HRQoL, as determined by Short Form 36 (SF-36) among patients after TIA or ischemic stroke one-year following their initial entry in hospital.

**Methods:**

Patients were assigned to either an intervention (IG) or a control group (CG). The individual assignment of the patient to IG or CG depended on the community pharmacy to which the patients were assigned for care. Community pharmacies either delivered standard care (CG) or provided intensified PC (IG). Pharmacists who are members of the "Quality Assurance Working Group" (QAWG) provided PC for patients in IG.

**Results:**

255 patients were recruited (IG: n = 90; CG: n = 165) between 06/2004 to 01/2007. During the study, the HRQoL of the patients in IG did not change significantly. In the CG, a significant decrease in the HRQoL was observed in 7/8 subscales and in both summary measures of SF-36.

**Conclusions:**

This is the first follow-up study in Germany involving a major community hospital, rehabilitation hospitals, community pharmacies and general practitioners investigating the impact of PC on HRQoL of patients after ischemic stroke. Our findings indicate that an intensified education and care of patients after ischemic stroke by dedicated pharmacists based on a concept of PC may maintain the HRQoL of IG patients.

## Background

Stroke is one of the leading causes of death in Europe and the major cause of disability in the elderly [1,2]. Health-related quality of life (HRQoL) related to stroke and life satisfaction after stroke are important healthcare outcomes that have not received sufficient attention in the literature. HRQoL assessment includes at least 4 dimensions: physical, functional, psychological, and social health [3,4]. The physical health dimension refers to disease-related symptoms. The functional health comprises self-care, mobility, and the capacity to perform various familiar and work roles. The psychological dimension includes cognitive and emotional functions and subjective perceptions of health and life satisfaction. The social dimension includes social and familiar contacts [3]. Short Form 36 is a widely and frequently used, generic, patient-report instrument for measuring quality of life [5]. It has also been validated in patients with stroke [5,6].

Pharmaceutical Care (PC), first outlined by Hepler and Strand in 1990, has been the subject of intensive research in Germany for several years. PC is the provision of drug therapy by a responsible pharmacist for the purpose of achieving a definite outcome to improve the patients' quality of life [7]. PC is a patient-tailored, outcome oriented pharmacy practice that requires that the pharmacist work in concert with the patient and the patient's other healthcare providers to promote health, to prevent disease, and to assess, monitor, initiate, and modify medication use to assure that drug therapy regimens are safe and effective. Thus, PC is a concept to optimize drug therapy, minimize drug-related problems, and improves self-management; it can directly affect the HRQoL of patients. The pharmacist is a part of the health care team, and extensive communication between pharmacist, physician, and the patient is necessary to achieve a defined health care outcome [7]. The goal of Pharmaceutical Care is to optimize the patient's HRQoL, and achieve positive clinical outcomes, within realistic economic expenditures. The positive influence of PC on HRQoL has been demonstrated in several trials [8,9].

The main objective of this study was to evaluate the impact of PC on the HRQoL, as determined by Short Form 36 (SF-36) among patients after TIA or ischemic stroke one-year following their initial entry into the hospital.

## Methods

### Setting

A major community hospital in Germany (Klinikum Fulda gAG), several rehabilitation hospitals, community pharmacies and general practitioners were involved in this study. The Stroke Unit of the Klinikum Fulda gAG serves diagnosis and treatment of over 700 patients with acute stroke each year.

### Patients

Patients with TIA or ischemic stroke with a Barthel index of over 30 points at the time of discharge from the hospital and living at home were included between June 2004 and January 2007. The follow-up-period for the patients was 12 months. Demographic and clinical data were collected from medical records and by interview.

The patients were assigned either to an intervention group (IG) or a control group (CG). The individual assignment of the patient to the IG or CG depended on the community pharmacy to which patients were assigned for care. Pharmacists (n = 23) who are members of the "Quality Assurance Working Group" (QAWG) provided PC for patients in the IG. Pharmacists of the QAWG met on a regular basis (once a month) to discuss specific drug-related issues and to ensure proper implementation of PC in their pharmacies. The PC process in this study comprises medication reviews and counselling interviews with regards to medicines, especially those for secondary prevention, and to specific actions, side effects, and drug-interactions as well as cardiovascular risk factors such as hypertension, diabetes mellitus, and hyperlipidemia. *Hypertension *remains a major modifiable risk factor for cardiovascular diseases. The diagnosis of hypertension is made when the average of two or more diastolic blood pressure (BP) measurements on at least two subsequent visits is more than 90 mm Hg or when the average of multiple systolic BP readings on two or more subsequent visits is consistently more than 140 mm Hg [10]. *Diabetes mellitus *is a group of metabolic diseases characterised by chronic hyperglycemia resulting from defects in insulin secretion, insulin action, or both [11]. *Hyperlipidemia *is defined as an abnormal plasma lipid status. Common lipid abnormalities include elevated levels of total cholesterol, low-density lipoprotein (LDL) cholesterol, lipoprotein (a), and triglyceride; as well as low levels of high-density lipoprotein (HDL) cholesterol. These abnormalities can be found alone or in combination [12]. Furthermore, the pharmacists had to detect and solve drug-related problems (DRPs). The community pharmacists received education and training in PC with respect to patients with stroke in several workshops. Topics included causes of stroke, risk factors, symptoms, definition of PC, identifying and solving DRPs and designing a PC plan. Risk factors such as hypertension, diabetes mellitus, hyperlipidemia, and atrial fibrillation as well as secondary prevention and individual patient problems were discussed. The other community (control) pharmacists (n = 39) delivered standard care for the patients within the CG.

### Pharmaceutical Care

The PC process for the patients in the IG consists of three sections:

(i) *At hospital*: Patients and their relatives received a counselling interview from the clinical pharmacist about the current medication, their effects, the dosage and important side effects as well as administration advice. A medication record with detailed information about the drug name, the dosage and administration advice was provided.

(ii) *Seamless care*: At the time of discharge from hospital, the general practitioner, the rehabilitation hospital, and the community pharmacist received a detailed care plan for the patient from the clinical pharmacist.

(iii) *In the ambulatory setting*: PC was continued by the attending pharmacist for 12 months during this study. At least one counselling interview between the pharmacist and the patient every three months was obligatory. The attending pharmacist was required to document the medication, the DRPs and the counselling interview.

### Standard Care

Patients of the CG received a medication record at the time of discharge from hospital and received standard care from their community pharmacy. There is no defined standard care process for the community pharmacy and it depends on each pharmacist. General issues are for example dispensing medicine, giving advice on medicine, and information about side effects and drug-interactions.

**Figure 1:**
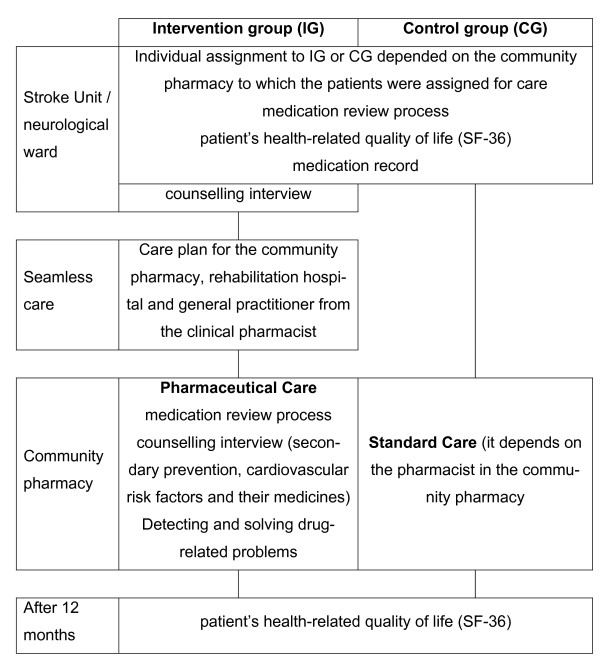
**The sequence and structure in the intervention and control group for 12 months**.

The steps taken over 12 months of the study are summarized in Figure 1.

### Health-related quality of life

To evaluate the patient's HRQoL, the SF-36 was used upon entry into the hospital and after 12 months. It provides a valid, subjective measure of physical and mental health after stroke. Two types of SF-36 scores can be generated: the 8 SF-36 scales provide a comprehensive profile of health status, and the two summary measures have features that make them more advantageous for clinical trials [5]. The SF-36 consists of 8 subscales: physical functioning (PF), physical role (RP), bodily pain (BP), general health (GH), vitality (VT), social functioning (SF), emotional role (RE), and mental health (MH), and the 2 summary measures are the Physical Component Summary (PCS) and the Mental Component Summary score (MCS).

Subscales and the summary scores range from 0 to 100, with higher values representing better quality of life.

Different sociodemographic and medical variables were recorded, including age, sex, cerebrovascular risk factors, the type of cerebral ischemia (transient ischemic attack (TIA), or ischemic stroke), and the Barthel Index.

The study was conducted in compliance with the requirements of the institutional review board, Philipps University, Marburg (Germany). All patients signed the informed consent.

### Statistical Analysis

All statistical analyses were performed by SPSS version 12.0 for Windows. Sample size calculations targeting a power of 80% (2-sided test of α = 5%) to improve the patient's HRQoL after stroke on the basis of PC demonstrated that 116 patients were needed for each group.

The evaluation of the HRQoL was based on a "per-protocol" analysis; only those patients were analyzed who completed the entire trial.

Data are shown as means. The chi-squared test was used for assessing differences in proportions. The students t-test was used to compare the means of both groups. In order to determine the normal distribution of the variables the Kolmogorov-Smirnov-test was used. The non-parametric rank-sum test according to Mann and Whitney was used to compare the distribution of ranks between the groups. The Wilcoxon Rank sum test was chosen to identify differences in each group at baseline and after 12 months. A two-tailed probability value of <0.05 was considered to be statistically significant.

## Results

**Figure 2:**
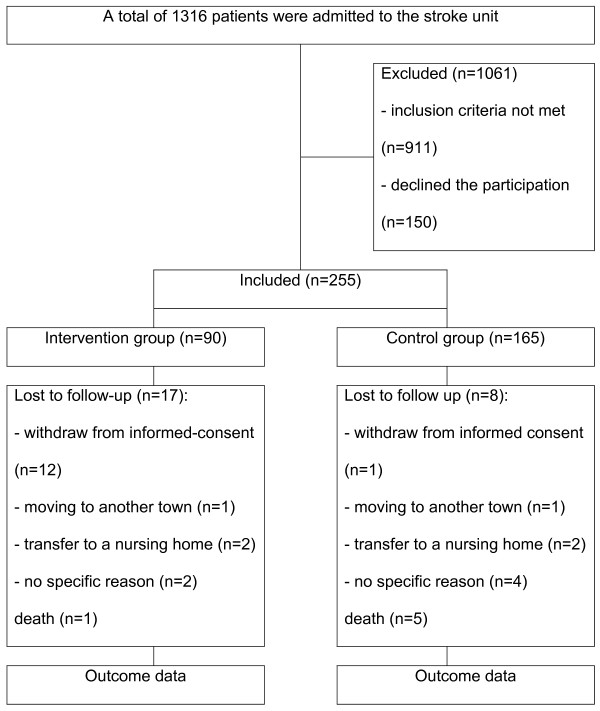
**Flow chart for patients' inclusion and exclusion criteria and their participation**.

Over the entire period of enrolment (20 months), 1316 patients were admitted to the Stroke Unit. In total, 911 patients (69.2%) were excluded from the study due to intracranial haemorrhage or other diagnoses, for example migrainous aura, seizures, Barthel index < 30 points at discharge from hospital, fatal ischemic stroke or living in a nursing home. A total of 405 patients met the inclusion criteria, 150 patients of these (37.0%) declined participation in the study. Reasons for refusal were an excellent support by the family or general practitioner, no need for further information or intensive care, or the patient was not familiar with PC. Of the remaining 255 patients, n = 90 were recruited into IG and n = 165 into CG (Figure 2).

Patients' baseline characteristics are summarized in Table 1. Between IG and CG, there were no significant differences with regards to age (p = 0.952), sex (p = 0.554) or subtype of cerebral ischemia (p = 0.814), and Barthel Index (p = 0.101). The most frequent cardiovascular risk factor in both groups was arterial hypertension (37%); the second most frequent was a combination of hypertension and hyperlipidemia.

**Table 1:** Baseline characteristics

Characteristics	Intervention Group n = 90	Control Group n = 165
Age, mean ± SD (years)	68.2 (± 9.7)	68.1 (± 10.8)
Min	45	23
Max	85	85

Sex, n (%)		
female	35 (38.9%)	58 (35.2%)
male	55 (61.1%)	107 (64.8%)

subtype of cerebral ischemia, n (%)		
TIA	28 (31.1%)	49 (29.7%)
Ischemic stroke	62 (68.9%)	116 (70.3%)

Barthel Index, mean ± SD	92,8 (± 7,5)	89,4 (± 14,3)

cardiovascular risk factors		
hypertension	33 (36.7%)	61 (37.0%)
hyperlipidemia	6 (6.7%)	5 (3.0%)
diabetes mellitus	1 (1.1%)	2 (1.2%)
hypertension+hyperlipidemia	31 (34.4%)	46 (27.9%)
hypertension+diabetes mellitus	4 (4.4%)	20 (12.1%)
diabetes mellitus+hyperlipidemia	--	2 (1.2%)
hypertension+diabetes		
mellitus+hyperlipidemia	7 (7.8%)	15 (9.1%)
None	8 (8.9%)	14 (8.5%)

SD = Standard deviation

During the study period 17 patients (18.9%) in the IG and 8 patients (4.9%) in the CG were lost during follow-up. Reasons for patients' drop-out were withdrawal from informed consent, moving of the patient to another town, transfer to a nursing home or no specific reasons. One patient in the IG and five patients in the CG died (Figure 2). No differences in sex, ages, and diagnosis were found in both groups at time of discharge from hospital.

### Health-related quality of life

#### Eight subscales

**Figure 3:**
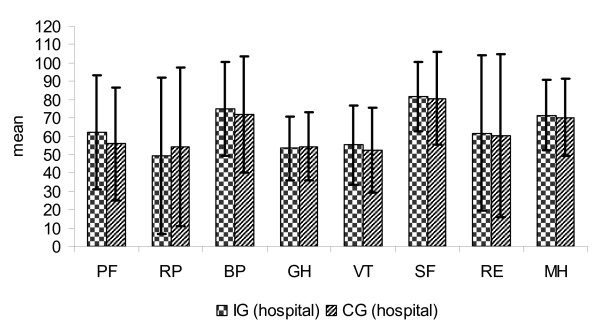
**Score distribution of the SF-36 (8 subscales) IG and CG at hospital (mean and standard deviation)**.

**Figure 4:**
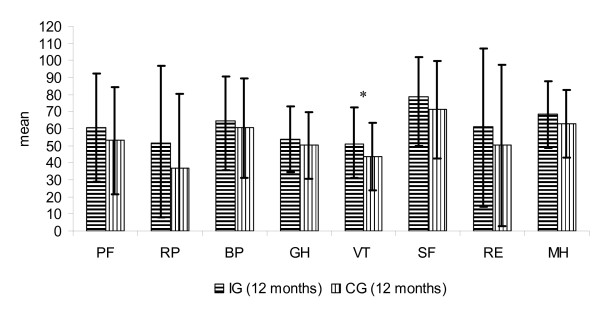
**Score distribution of the SF-36 (8 subscales) IG and CG after 12 months (mean and standard deviation)**.

**Figure 5:**
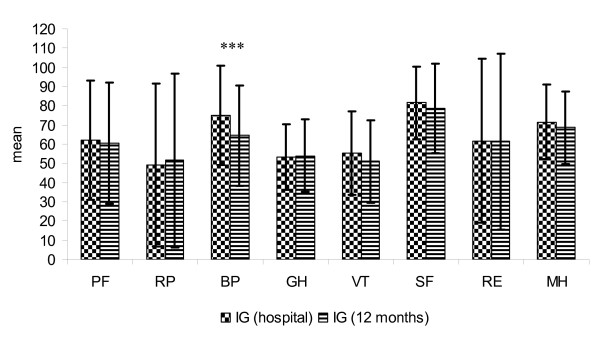
**Score distribution of the SF-36 (8 subscales) IG in hospital and after 12 months (mean and standard deviation)**.

The distribution of SF-36 scores in the IG (n = 64) and in the CG (n = 119) is shown in Figure 3, 4, 5, 6. Upon admission patients of both, IG and CG, groups showed no significant differences in the 8 subscales (Figure 3). After 12 months, the subscale *vitality *was significantly lower in CG patients than in IG patients (p = 0.027) (Figure 4).

**Figure 6:**
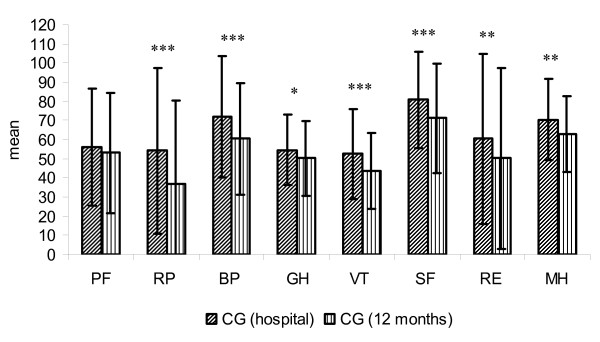
**Score distribution of the SF-36 (8 subscales) CG in hospital and after 12 months (mean and standard deviation)**. PF: physical functioning. RP: physical role. BP: bodily pain. GH: general health. VT: vitality. SF: social functioning. RE: emotional role. MH: mental health. * = p < 0.05. ** = p < 0.01. *** = p < 0.001.

The HRQoL of patients in IG remained stable over the entire observation period. Only the subscale *bodily pain *decreased significantly over time (p = 0.001) (Figure 5). In contrast, HRQoL parameter of patients in CG deteriorated significantly in 7 of the 8 subscales over time (Figure 6).

#### Two summary measures

The mean scores of both summary measures, PCS and MCS, are shown in Table 2. Upon entry into the study there was no significant difference in the two summary measures in either group. At follow-up, there were also no significant differences in HRQoL between the groups (Table 2). For the CG, a significant decline in PCS and MCS was observed between baseline and at follow-up (PCS: p = 0.023; MCS: p = 0.001). For the IG, changes in PCS and MCS between study entry and at follow-up were not statistically significant.

**Table 2:** Score distribution of the SF-36 (2 summary measures) at hospital and after 12 months (per-protocol-analysis)

			IG (n = 64)	CG (n = 119)	After 12 months Between group comparison
SF-36 Scales		time	mean (± SD)	mean (± SD)	p-Value †
PCS		Hospital	41.8 (± 9.8)	41.2 (± 10.8)	
		After 12 months	41.5 (± 11.0)	38.1 (± 11.6)	**0.090**
	**p-Value ‡**	Differences between baseline and after 12 months	**0.313**	0.023*	
					
MCS		Hospital	49.5 (± 10.6)	49.6 (± 11.7)	
		After 12 months	48.1 (± 11.3)	45.0 (± 10.9)	**0.077**
	**p-Value ‡**	Differences between baseline and after 12 months	**0.831**	0.001***	

SD = Standard deviation.

**† **= The Mann-Whitney U test was used to compare the distribution of ranks between the groups.

**‡ **= The Wilcoxon Rank sum test was chosen to identify differences in each group at baseline and after 12 months.

## Discussion

This is the first study in Germany to investigate the impact of intensive pharmaceutical care (PC) versus standard care in a larger patient population with ischemic stroke. This study demonstrates that PC is able to prevent deterioration of most HRQoL parameters over a 12 months period. Our findings indicate that PC as performed in the hospital and in the community setting is feasible and has a clear benefit and positive impact on patient's HRQoL. Moreover, this study is a step forward towards monitoring the implementation of PC within hospitals and within the community setting and towards evaluating the role of pharmacists within a specific therapeutic team. We found that a PC concept stabilizes the HRQoL in several scales as a result of an intensified involvement by pharmacists.

In a comparison of the subscales and the summary measures in both groups upon study entry there were no significant differences, thus providing an optimal starting point for measuring the impact of PC on HRQoL. After 12 months the comparison of the HRQoL of the patients who received intensified PC with those who received standard care showed that the vitality of the patients in CG was significantly lower than of patients in IG. The other subscales and the summary measures showed lower, but not significant, HRQoL scores in CG indicating inferior HRQoL as compared with the patients in IG. Due to ethical considerations community pharmacies were not restricted from delivering PC as it did not seem appropriate to keep the control pharmacists from providing PC in the case that DRP became apparent. For this reason the comparison may not to be statistically significant.

Most importantly, there was a significant deterioration of 7 of 8 subscales as well as both summary measures of the HRQoL over the 12 months period in the CG. That means that patients who do not receive intensified PC have a higher chance of deteriorating HRQoL. For the IG, there was only a significant deterioration in *bodily pain *at follow up. The deterioration of the HRQoL in patients with cerebrovascular diseases has been reported in previous trials in several parameters [3,13,14]. The quality of the patient's recovery can be evaluated on the basis of the activity of daily living (ADL), social activities, and return to work. Independently of ADL significant deleterious effects in HRQoL result from continuing loss of function due to the stroke. The deterioration in the HRQoL can be caused by the presence of anomalous perception and dissatisfaction in patients with minor disability levels that were incompletely restored after the stroke. Thus, the consequences of mild to moderate stroke can affect all dimensions of HRQoL despite the patient achieving full independence as measured in ADL [3].

By participating in this study the HRQoL of the patients in the IG was stable in several subscales and both summary measures indicating that the HRQoL can be maintained by intensified PC. Several studies have also demonstrated that PC may have a positive impact on the patients' HRQoL for example in patients with asthma or hypertension [8,15,16]. Graesel et al. investigated the impact of an intensified transition concept between inpatient neurological rehabilitation and home care of patients with stroke. The intensified transition concept included four additional elements: a psycho-educational seminar for family carers; an individual training course for carers; therapeutic weekend care at home before discharge; and finally, telephone counselling three months after discharge. The results show that this concept did not lead to any significant benefit, neither to functional status nor to HRQoL [17]. This finding may indicate that the standard transition already contains effective elements of preparation for home care and that therefore no further decisive advantage can be attained. Otherwise the number of patients (n = 62) as well as the observation period (n = 6 months) in the study was limited, thus excluding long-term effects.

The HRQoL of the patients in the CG decreased significantly in the subscales and in both summary measures. The deterioration of the physical role was significant while physical functioning did not significantly deteriorate, which may indicate that patients perceived a decline in their physical conditions.

There is a large difference in the frequency of diabetes mellitus in both groups (IG: n = 12 (= 13.3%); CG: n = 39 (= 23.6%)). Diabetes mellitus has detrimental effects on a range of health outcomes including HRQoL; for example people with diabetes have lower scores at the SF-36 [18]. Thus, the higher proportion of patients with diabetes in the CG may possibly cause the deterioration of HRQoL.

It should be pointed out that a considerably higher proportion of subjects in the IG than in the CG refused to participate. Those patients who refused to participate had comparable demographic data, but may have had more medical, social or psychological problems which could have affected the HRQoL. The difference in HRQoL after one year could be due to selection bias.

The mortality rate in both groups seems to be remarkably low (IG: n = 1; CG: n = 5); which is due to the fact that patients with a Barthel index of less than 30 points at discharge from hospital and/or who live in a nursing home were excluded from this study. These exclusion criteria were defined because only patients mobile enough to go the pharmacy can be given PC.

An important difficulty in the analysis of HRQoL in stroke patients is that few studies use the same measurement tools and the same period of follow up. Furthermore, there are no data in the literature from the patients' HRQoL from before the cerebrovascular event.

### Limitations

The limitations of the present study include the fact that no blinding or randomization could be performed. A target number of 116 participants were calculated for each group. However, only 90 patients could be recruited for the IG, as many patients refused to participate. The time of enrolment was extended from 12 to 20 months and then stopped. Moreover, patients who were lost to follow-up were not evaluated for HRQoL, because no follow-up data were available. It should be pointed out that, due to ethical considerations, it did not seem to be appropriate to keep the control pharmacists from caring in the case that DRP became apparent. Furthermore, we did not have any information on socioeconomic status, financial resources or caregiver support in patients of either group, which could have influenced the outcome of the patients.

## Conclusions

In conclusion, this is the first follow-up study in Germany involving a major community hospital, rehabilitation hospitals, community pharmacies and general practitioners demonstrating the potential impact of intensified pharmaceutical care for patients with ischemic stroke on HRQoL. Our findings indicate that an intensified education and care of patients after ischemic stroke by dedicated pharmacists based on a concept of PC may have a positive impact on HRQoL.

## Competing interests

The authors declare that they have no competing interests.

## Authors' contributions

CH has made substantial contributions to conception and design, acquisition of data, analysis and interpretation of data. RR, and JMK have made substantial contributions to conception and design. All authors have been involved in interpretation of data, drafting the manuscript or revising it critically for important intellectual content and have given final approval of the version to be published.
